# Efficient visible light modulation based on electrically tunable all dielectric metasurfaces embedded in thin-layer nematic liquid crystals

**DOI:** 10.1038/s41598-019-45091-5

**Published:** 2019-06-17

**Authors:** Mingyu Sun, Xuewu Xu, Xiao Wei Sun, Xin’an Liang, Vytautas Valuckas, Yuanjin Zheng, Ramón Paniagua-Domínguez, Arseniy I. Kuznetsov

**Affiliations:** 1 School of Electrical and Electronic Engineering, Nanyang Technological University, 50 Nanyang Avenue, 639798 Singapore, Singapore; 2 Institute of Materials Research and Engineering, A*STAR (Agency for Science, Technology and Research), 2 Fusionopolis Way, 138634 Singapore, Singapore; 3 Department of Electrical and Electronic Engineering, College of Engineering, South University of Science and Technology of China, 1088 Xue-Yuan Road, Shenzhen, Guangdong Province 518055 China

**Keywords:** Metamaterials, Liquid crystals

## Abstract

All-dielectric metasurfaces have attracted attention for highly efficient visible light manipulation. So far, however, they are mostly passive devices, while those allowing dynamic control remain a challenge. A highly efficient tuning mechanism is immersing the metasurface in a birefringent liquid crystal (LC), whose refractive index can be electrically controlled. Here, an all-dielectric tunable metasurface is demonstrated based on this concept, operating at visible frequencies and based on TiO_2_ nanodisks embedded in a thin LC layer. Small driving voltages from 3~5 V are sufficient to tune the metasurface resonances, with an associated transmission modulation of more than 65%. The metasurface optical responses, including the observed electric and magnetic dipole resonance shifts as well as the interfacial anchoring effect of the LC induced by the presence of the nanostructures, are systematically discussed. The dynamic tuning observed in the transmission spectra can pave the way to dynamically tunable metasurface devices for efficient visible light modulation applications.

## Introduction

Metasurfaces are two-dimensional spatial arrangements of sub-wavelength scatters engineered to manipulate the incoming wavefront^1^. They are used to realize different functionalities ranging from flat lenses^2–6^, polarization control^7–10^ or light bending^11–15^ to hologram reconstruction^16–19^ or complex beam shaping^20–24^. While the field of metasurfaces started developing based on metallic components, the low efficiency of those devices for short wavelength, mainly due to Ohmic dissipation and scattering losses, made the scientific community turn its attention to all-dielectric alternatives^25,26^. In the particular case of metasurfaces using high index dielectric nanoantennas, they not only benefit from the lower Ohmic losses, but also from the fact that these nanostructures naturally support a rich phenomenology of optical modes, including both electric and magnetic multipolar Mie resonances, which offer a great flexibility to tailor their scattering directivity^27^.

Most of the metasurfaces demonstrated so far are static. However, applications in display and other optoelectronic device industries require electrically switchable and compact optical components. To dynamically control the modulation of light by a metasurface, several strategies have been explored. At microwave frequencies, one can do that efficiently by using diodes^28^. At optical frequencies, where this strategy cannot work, one can *e*.*g*. introduce mechanically deformable meta-atoms or substrates and possibly use MEMS to tune their resonances^29–33^ or change the dielectric environment of the metasurface using birefringence crystals^34^, graphene^35^, gated semiconductors^36^, phase change materials^37^ or electro-optical polymers^48^ to name a few. However, the tuning range for the cases of charge injection or electro-optical effects is limited due to the small permittivity change, particularly at high frequencies^36,38^, while phase change materials tend to be lossy in the visible spectral range^37^. Liquid crystals (LC), have a large birefringence (∆*n*~0.2) at room temperatures and are almost lossless in the visible range. This, together with the mature LC technology, renders them an ideal platform for active devices for the visible spectral range addressable via electrical, optical, magnetic and thermal approaches^39^. Recently, several works reported tunable devices by combining LCs with plasmonic^40,41^ and dielectric^34,42–46^ metasurfaces. So far, most of the LC-based high-index dielectric metasurfaces reported used silicon as meta-atoms, showing large tunability of the supported electric and magnetic dipole resonances in the near-IR frequency range under external thermal^34,43,44^ and electrical control^45^. However, considering the low band gap of silicon (*E*_g_ ≈ 1.1 eV), significant losses are unavoidable for such metasurfaces in the visible spectral range (*hν* > 1.6 eV). In the meantime, the systematic discussion on evolution of electromagnetic resonance within metasurface nanoantennas with the LC birefringence is still insufficient.

In this paper, we demonstrate a transmission-type, LC-based tunable metasurface based on titanium dioxide (TiO_2_) nanoantennas efficiently operating in the visible spectral range. TiO_2_ has a wide band gap (*E*_g_ ≈ 3.2 eV), thus being lossless across the entire visible spectrum^13^. To achieve resonance tuning, the TiO_2_ nanodisks were embedded in a thin, 1.5 µm, LC cell. We show dynamic control of the electric and magnetic resonances by switching the LC alignment under electrical voltages. We also study the influence of the anchoring effect induced by the nanoantennas on the LC anisotropy through theoretical and experimental investigations, which is related to the morphology and material composition of the meta-atoms, affecting the LC molecule alignment direction.

## Results and Discussion

### Description of sample fabrication and characterization

**Figure 1:**
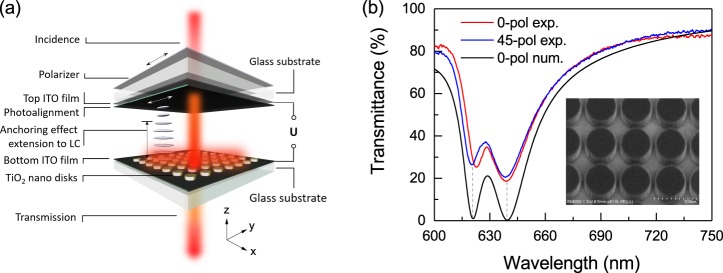
(**a**) Sketch of electrically tunable transmission type TiO_2_ metasurface with LC infiltration sandwiched by ITO-coated glass substrates. The LC, in the absence of any applied voltage, is aligned in the *x*-axis by the photoalignment layer coated onto the top ITO glass surface (the photoalignment direction is given by the white arrow). LC alignment in the vicinity of the nanostructures can be altered by its presence influenced by both the photoalignment layer and the anchoring effect of nanostructures; (**b**) Comparison of calculated (*P* = 360 nm, *D* = 320 nm and *H* = 188 nm) (black curve) and measured (red and blue curves) transmittance spectra of the TiO_2_ nanodisks metasurface before the LC infiltration. The inset gives the scanning electron microscope (SEM) image of the fabricated TiO_2_ metasurface.

A schematic illustration of the transmission-type LC based TiO_2_ dielectric metasurface device is shown in Fig. 1a. The metasurfaces were fabricated from 190 nm thick amorphous TiO_2_ films deposited on commercially available ITO-coated soda lime glass substrates using electron-beam lithography (EBL) followed by inductively-coupled plasma reactive ion etching (ICP-RIE). They were then sandwiched with another ITO-coated glass substrate and infiltrated with a 1.5 µm-thick LC layer in vacuum. The commercial liquid crystal E7 from Merck® was used, which is characterized by a strong room temperature birefringence between the extraordinary and ordinary refractive indices (∆*n* = *n*_e_ − *n*_o_ ≈ 0.2). The appropriate thickness of the LC cell was obtained using commercially available tiny silica spheres as the spacer. The reason for using very thin LC cells is to reduce crosstalk between the neighbouring nanoantennas to allow single pixel switching in the future. The TiO_2_ patterned nanodisks have the diameter *D* = 320 nm and are arranged in a square lattice with a periodicity *P* = 360 nm (see the inset in Fig. 1b). To achieve the high refractive index change of the LC by applying electrical voltage, one should first prepare initial, so-called nematic, LC state with all molecules aligned parallel to the light polarization direction. This can be achieved by either mechanical rubbing or photo-induced alignment of polymer molecules deposited on the LC cell surface. It has been previously reported that both the surface roughness and the static electricity may strongly influence the rubbing-induced alignment of thin LC cells^47^. Thus, instead of the rubbing, we chose the photoalignment process based on the *azo dye* solution coated on the top LC cell layer for LC nematic state alignment (see Fig. 1a). The fabrication of *azo dye* solution is given in detailed in the Methods section. We pre-set the initial alignment direction of LC along the *x*-axis as shown in Fig. 1a. The detailed description of both the TiO_2_ metasurface fabrication and the LC infiltration process is given in the *Methods Section*.

Optical transmission spectra of the fabricated sample were characterized using a customized inverted micro-spectrometry setup described in detail elsewhere^48^. Details of the optical measurement are described briefly in the *Methods* section. The measured transmittance of the fabricated TiO_2_ metasurface before the LC infiltration is given in Fig. 1b. In the measured results, two resonance dips can be clearly seen at *λ* ≈ 620 nm and *λ* ≈ 640 nm corresponding, respectively, to the electric dipole and magnetic dipole resonances supported by the nanodisks in the metasurface. Good correspondence is found with numerical simulations performed using a Finite-Difference Time-Domain commercial software (Lumerical FDTD, see the *Methods Section* for details) with the diameter *D* = 320 nm and height *H* = 188 nm in the periodicity *P* = 360 nm, almost close to the designed geometry. These resonance positions are almost identical even when the incident polarization is rotated by 45°, as shown in the graph, due to the symmetry in the metasurface.

### Optical response of the metasurface after LC infiltration

**Figure 2:**
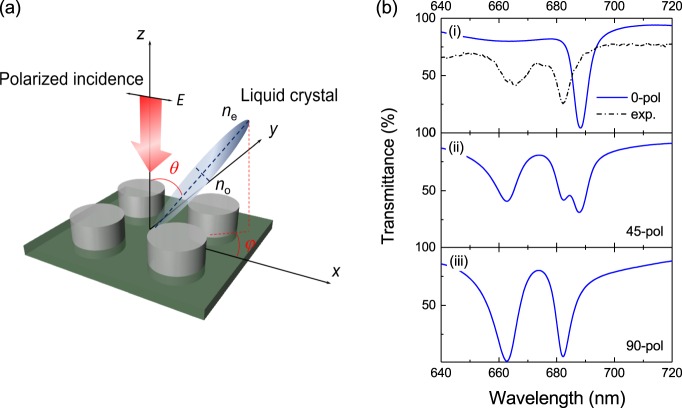
(**a**) Sketch of the LC alignment model. Polar angle *θ* and azimuthal angle *φ* define the spatial alignment of LC molecules with respect to the *x*-axis corresponding to the photo-alignment direction of the LC cell. The alignment direction is defined by the long axis of LC molecules. (**b**) Calculated transmittance spectra of the TiO_2_ nanodisk metasurface after LC infiltration for the incident light polarized in-plane along (i) *φ* = 0°, (ii) *φ* = 45° and (iii) *φ* = 90° directions, with LC alignment set in-plane parallel to the x-axis (*θ* = 90°, *φ* = 0°). The dashed line shows the experimental results for incident polarization parallel to *φ* = 0°.

The optical response of the metasurface after the LC infiltration was first numerically simulated to investigate the influence of the anisotropic environment on its resonant behaviour. The details of the numerical simulations are given in *Methods Section*. The geometry and parameters of the TiO_2_ nanodisks are kept according to those specified above (see Fig. 1). We denote as LC alignment to the spatial orientation of the LC molecules’ directors (see Fig. 2a), being defined as the direction of the long axis of the LC molecules. We start by considering that the LC alignment is set in-plane, parallel to the photoalignment direction along the *x*-axis (polar angle *θ* = 90° and azimuthal angle *φ* = 0°), thus characterized by an optical anisotropy defined by the extraordinary *n*_x_ = *n*_e_ and ordinary *n*_y_ = *n*_z_ = *n*_o_ refractive indices. Transmittance spectra of the array calculated for in-plane incident light polarization parallel to *φ = *0°, *φ* = 45° and *φ* = 90° directions are given in Fig. 2b. The electric and magnetic dipole resonances were identified by recording the electric and magnetic fields at the center of the nanoantenna using a point monitor and analyzed in Supplementary Figure S1. For the incident polarization along the *x*-axis (*φ* = 0° incidence), the electric and magnetic dipole resonances are almost overlapped at *λ* ≈ 688 nm, which explains the single dip observed in the transmission spectrum. For the incident polarization along the *y*-axis (*φ* = 90° incidence), the electric resonance blue shifts to *λ* ≈ 662 nm, while the magnetic resonance only slightly shifts to *λ* ≈ 682 nm and thus two dips in transmission are observed. The observed blue shifts of the resonances for the orthogonal polarization can be attributed to the reduced refractive index from *n*_e_ to *n*_o_ along the polarization direction. Comparatively, the magnetic resonance shows less sensitivity to the refractive index change than the electric one. This effect is known to be related to a higher confinement of the magnetic dipole mode inside the nanoparticle^48,49^ and thus its lower sensitivity to the environment change. By analyzing the electric and magnetic dipole resonances separately (see Supplementary Figure S1), one can see a co-existence of two electric dipole resonances at *λ* ≈ 662 nm for *n*_o_ and *λ* ≈ 688 nm for *n*_e_ during the incident polarization rotation from *φ* = 0° to 90°. The component ratio between the two electric dipoles depends on the rotation angle of incident polarization. To be noted, an obvious splitting of magnetic mode is also observed for incident polarization angles between *φ* = 15° and 75°. This can be explained by the anisotropy of the LC. In polarization states having components both along the ordinary and the extraordinary axes, two different dipolar resonances are excited with spectral positions corresponding to the different refractive indices felt in the anisotropic optical environment^50^. For the *φ* = 45° polarized incidence, the excitation components along the *x* and *y*-axis become nearly equivalent and, thus, the transmittance spectrum shows an obvious combination of those observed under *φ* = 0° and 90° polarized incidence, further corroborating the influence of the anisotropic optical environment with respect to the two directions (see Supplementary Figure S1b). The higher transmittance depth at the resonances for *φ* = 45° incident polarization should come from the certain amount of transmission due to the rotation of LC.

For the fabricated TiO_2_ metasurface, the transmission spectra after LC infiltration were measured at room temperature and compared with the simulation results. For that, we first confirm that the LC is correctly aligned along the *x*-axis. We do so by measuring transmitted light through the bare LC (i.e. far away from the nanostructures) when the incident polarization is rotated from *φ* = 0° to 90° and the analyzer is perpendicularly oriented. The results (shown in Supplementary Figure S2a) show maximum transmission for *φ* = 45° and vanishing one for *φ* = 0° and *φ* = 90° confirming the correct alignment of the LC along the *x*-axis. The transmittance through the metasurface was also measured under various incident polarizations and compared to the numerical simulations. As shown in Fig. 2b (i), this comparison gives a very poor agreement between measured and simulated results for 0° incident polarization. This indicates a strong misalignment of the LC molecules with respect to the LC cell photoalignment direction on top of the metasurface (i.e. in the presence of the nanostructures), which motivates the study presented in the next section.

### Refined model incorporating LC anchoring effects with nano antennas

**Figure 3:**
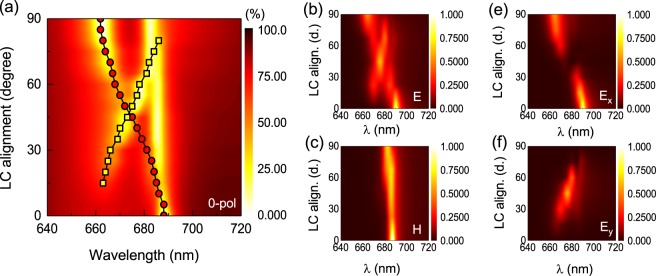
(**a**) Calculated transmittance spectra of TiO_2_ nanodisks metasurface after LC infiltration for variation of in-plane LC alignment angles ranging from *φ*  =  0° to 90° with respect to the photoalignment direction (the *x*-axis) using the parallel (*pa*.) alignment model. The incident light is polarized along the *x*-axis (0° pol.); (**b**) electric and (**c**) magnetic dipole resonances and the excited electric dipole components along the (**e**) *x* and (**f**) *y* directions for the incident polarization parallel to *φ* = 0° with LC in-plane alignment varied from *φ*  =  0° to 90° to the *x*-axis corresponding to (a).

It has been previously reported that LCs may preferentially align along a certain direction defined by the surface energy of nanostructures^51^. This, so called, anchoring effect of LC can be expected to significantly affect the LC alignment near the nanoantennas, introducing in-plane deviations in the LC alignment from the preset rubbing or photoalignment direction^34^. Considering the combined effect of the photoalignment and anchoring, we propose two models, which we name the parallel (*pa*.) and twisted (*tw*.), to simulate the LC alignment (see *Methods Section* and Supplementary Figure S3). In both cases, we consider that, near the nanodisks, the LC is aligned to a certain direction given by an azimuth angle *φ* with respect to the initial photoalignment direction, as a result of the anchoring effect. For the *pa*. model, the LC molecules retain the same direction across the whole LC cell from the nanoantennas to the top layer. This represents the case of strong anchoring effect with a large extension distance. For the *tw*. model, the LC continuously twists across the cell from the given direction near the nanoantennas to being fully parallel to the photo-alignment direction (the *x*-axis) at the top layer, indicating a balanced competition between the anchoring effect by the nanoantennas and the alignment induced by the photoalignment layer. The calculated transmittance using both models with various azimuthal angles of LC alignment on top of the nanoantennas from *φ* = 0° to 90° is shown in Fig. 3a for *pa*. model and *x*-polarized light only, as well as the Supplementary Figure S4 for the whole set.

In the parallel model, the anisotropic effective refractive index can be approximately expressed as *n*_x_ = *n*_o_ + Δ*n*·cos*φ*, *n*_y_ = *n*_o_ + Δ*n*·sin*φ* and *n*_z_ = *n*_o_ (see the definition of *φ* in Fig. 2a). As it can be seen from Fig. 3b, the electric dipole resonant response along the *x*-axis (*E*_x_) continuously blue-shifts with the LC alignment rotation from *λ* ≈ 688 nm for *φ* = 0° to *λ* ≈ 662 nm for *φ* = 90° (circle-dotted curve in Fig. 3a). This can be explained by the continuous reduction of the refractive index along the excitation direction (the *x*-axis) from the extraordinary, *n*_e_, to the ordinary one, *n*_o_. The magnetic resonance, in turn, slightly shifts from *λ* ≈ 688 nm for *φ* = 0° LC alignment to *λ* ≈ 682 nm for *φ* = 90°. Interestingly, when LC alignment rotates to *φ* > 15°, the orthogonal electric resonance mode (*E*_y_, square dot curve in Fig. 3a) is observed and oppositely red shifted with LC rotation. The orthogonal component of the electric resonance appears due to the polarization rotation induced by the phase retardation associated with LC birefringence^41,52^.

For the twisted model (see Supplementary Figure S4d), the effective refractive index can be approximated as the integral accumulation of birefringence of each differential LC layer. In this case, the electric resonance blue shifts with the LC alignment rotation from *λ* ≈ 686 nm for *φ* = 0° to *λ* ≈ 670 nm for *φ* = 45°. Compared with the parallel model, the birefringence change due to LC alignment is normally smaller in twisted model. As the example shows for *φ* = 0° incident polarization, the *E*_x_ component of the electric dipole is more pronounced, while *E*_y_ is less evident for small LC rotation angles with respect to the *x*-axis, which helps to differentiate between the two models. Supplementary Figure S4 also shows these dependences on incident polarizations rotated by *φ* = 0°, 45° and 90° with respect to the *x*-axis.

**Figure 4:**
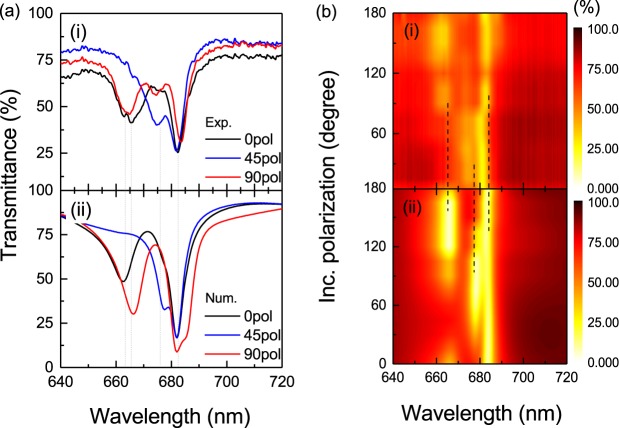
(**a**) Comparison of transmittance spectra after LC infiltration between (i) experimental and (ii) calculated results with *φ* = 30° LC initial alignment rotation for the parallel model for the incident polarization parallel to *φ*  =  0° (black curve), *φ*  =  45° (blue curve) and *φ*  =  90° (red curve); (**b**) The same comparison of (i) experimental and (ii) calculated results shown in 2D map for incident polarizations varied from 0 to 180°. The dash line marks the resonance positions.

We then compare the obtained refined LC model results with the measured values. After studying different calculated LC orientations for the two proposed models (Supplementary Figures S3 and S4) we conclude that the *pa*. model with LC molecules alignment at *φ* = 30° gives the best agreement to the experiment’s values (see Fig. 4). The near-field distribution is calculated for at the electric dipole and magnetic dipole resonances, and included in the Supplementary Figure S5, showing a large portion of the resonances localized within the disks. The non-zero resonance depth can be attributed to the possible reasons like the transmission due to LC misalignment, slit edge light scattering and off-normal incidence contributions. As it can be seen from the analysis of the excited electric and magnetic dipoles (see Supplementary Figure S6), the electric resonance along the *x*-axis (*E*_x_) excited at *λ* ≈ 675 nm is dominant for incident polarizations varied from *φ* = 15° to 90°. This corresponds to the higher effective refractive index for *φ* = 30° LC alignment. On the other hand, the electric resonance oriented along the *y*-axis excited at *λ* ≈ 664 nm dominates for incident polarizations varied from *φ* = 105° to 180° and corresponds to the lower effective refractive index.

The large deviation angle of LC in-plane alignment from the photoalignment direction indicates a strong anchoring effect in the thin LC layer, which dominates over the photoalignment influence. This effect can be considered based on the surface energy of the nanostructures, which is determined by the geometry of the metasurface and its symmetries. For the square array of nanodisks sample, due to symmetry considerations, the anchoring energy coefficients along the *x* and *y*-axis directions are taken to be equal. According to the Frank-Oseen model^51^, the system energy can be expressed as the sum of the elastic energy of LC (*F*_elas_) the surface anchoring energy (*F*_s_) and the photoalignment energy (*F*_ph_) as:

1 $$\begin{array}{ccc}{F} & = & {{F}}_{{\rm{e}}{\rm{l}}{\rm{a}}{\rm{s}}}+{{F}}_{{\rm{s}}}+{{F}}_{{\rm{p}}{\rm{h}}}\\  & = & {{F}}_{{\rm{e}}{\rm{l}}{\rm{a}}{\rm{s}}}+{({W}}_{1}\cdot {\sin }^{4}\phi +{{W}}_{1}\cdot {\cos }^{4}\phi )+{{W}}_{2}\cdot {\cos }^{4}\phi \\  & = & \frac{1}{2}\int \{{{K}}_{11}\cdot {({\rm{\nabla }}\cdot {\bf{n}})}^{2}+{{K}}_{22}\cdot {({\bf{n}}\cdot [{\boldsymbol{\nabla }}\times {\bf{n}}])}^{2}+{{K}}_{33}\cdot {({\bf{n}}\times [{\boldsymbol{\nabla }}\times {\bf{n}}])}^{2}\}{d}{V}\\  &  & +({{W}}_{1}\cdot {\sin }^{4}\phi +{{W}}_{1}\cdot {\cos }^{4}\phi )+{{W}}_{2}\cdot {\cos }^{4}\phi \\  & = & \frac{1}{2}{\int }_{0}^{d}[({{K}}_{11}{\sin }^{2}\theta +{{K}}_{33}{\cos }^{2}\theta )\cdot {({\theta }^{{\rm{^{\prime} }}})}^{2}+({{K}}_{22}{\sin }^{2}\theta +{{K}}_{33}{\cos }^{2}\theta )\cdot {\sin }^{2}\theta \cdot {({\phi }^{{\rm{^{\prime} }}})}^{2}]{d}{z}\\  &  & +{({W}}_{1}\cdot {\sin }^{4}\phi +{{W}}_{1}\cdot {\cos }^{4}\phi )+{{W}}_{2}\cdot {\cos }^{4}\phi \end{array}$$

where the *K*_11_, *K*_22_ and *K*_33_ are the splay, twist and bend elastic constants of LC, and *z* is the spatial coordinate integration variable, that extends along the thickness of the cell *d*.^39^ The coefficient *W*_1_ measures the strength of the anchoring energy of the nanostructures, and is the same, by symmetry considerations, along either *x* or *y* axes. *W*_2_ is the coefficient representing the strength of the photoalignment effect^40,51^. ***n*** represents the spatial LC director in terms of polar (*θ*) and azimuthal (*φ*) angles of alignment as ***n*** **=** (sin *θ* cos *φ*, sin *θ* sin *φ*, cos *θ*). A minimum system energy can be obtained according to the Euler-Lagrange equations for the system energy based on the parallel model of LC alignment at *φ* = 30° determined from experiments (*θ* = 90°, *φ*|_z=0_ = *φ*|_z=d_ = 30° and *φ*′|_z=0_ = *φ*′|_z=d_ = 0), as follows:

2 $$\begin{array}{c}\frac{{\rm{\partial }}{{F}}_{{\rm{e}}{\rm{l}}{\rm{a}}{\rm{s}}}}{{\rm{\partial }}\phi }-\frac{{{d}{F}}_{{\rm{e}}{\rm{l}}{\rm{a}}{\rm{s}}}}{{d}{z}}\cdot \frac{{\rm{\partial }}{{F}}_{{\rm{e}}{\rm{l}}{\rm{a}}{\rm{s}}}}{{\rm{\partial }}{\phi }^{{\rm{^{\prime} }}}}+\frac{{\rm{\partial }}({{F}}_{{\rm{s}}}+{{F}}_{{\rm{p}}{\rm{h}}})}{{\rm{\partial }}\phi }\\ ={{K}}_{22}\cdot {\phi }^{{\rm{^{\prime} }}}{|}_{z=0}+[{{W}}_{1}\cdot ({\sin }^{2}\phi {|}_{{z}=0}-{\cos }^{2}\phi {|}_{{z}=0})-{{W}}_{2}\cdot {\cos }^{2}\phi {|}_{{z}={d}}]=0\,\end{array}$$

This equation for the energy of anchoring effect *W*_1_ and photoalignment *W*_2_ leads to solution *W*_1_/*W*_2_=1.5, which implies a stronger influence of the anchoring effect from the nanostructures than that of the photoalignment layer in the performed experiments. This kind of anchoring effect in the presence of nanostructures for LC alignment may be critical for the design of LC based tunable metasurfaces and, therefore, should be considered when realizing practical devices based on those.

### Efficient optical modulation by electrical tuning of the metasurface spectral resonances

Finally, the electrical tuning performance of the device is quantified in experiments by measuring the change in the spectral transmission of the sample when a DC voltage is applied across the LC cell thickness. The tuning performance of the LC cell without the metasurface was first characterized in a cross-polarized geometry. For that, light intensity was measured after passing through the LC cell and an analyzer aligned perpendicularly to the incident polarization direction (Supplementary Figures S2b,c and S6). This experimental geometry allows measuring cross-polarized light induced by the LC cell birefringence. When no voltage is applied to the cell the transmittance is optimized for the incident light polarization along *φ* = 45° direction (Supplementary Figure S7a). However, the transmittance decreases from >80% to almost 0% when the applied voltage is increased above 3 V, indicating an almost complete switching of LC molecules to the vertical orientation (see Supplementary Figure S7b,c).

**Figure 5:**
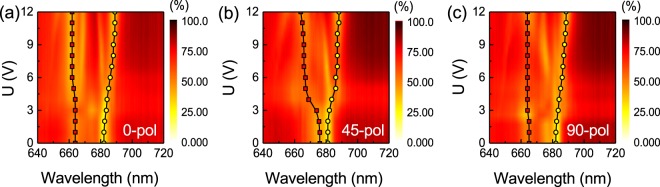
Experimental results of electrical tuning of the liquid crystal infiltrated TiO_2_ metasurface for the incident light polarization directions aligned at (**a**) *φ*  =  0°, (**b**) *φ*  =  45° and (**c**) *φ*  =  90° under the increased DC voltages from 0 to 12 V. The symbol-line curves mark out the movement of electric (red) and magnetic (yellow) resonance positions under the applied voltage.

The sample containing the metasurface was measured for three different incident polarizations, *φ* = 0°, 45° and 90°. The most pronounced resonance shifts are observed for incident polarization aligned at *φ* = 45° with respect to the *x*-axis, the closest to the predicted LC alignment including the anchoring effect. The electric resonance blue shifts from *λ* ≈ 675 nm to *λ* ≈ 664 nm and the magnetic resonance red shifts from *λ* ≈ 682 nm to *λ* ≈ 688 nm with applied voltages changing from 0 to 12 V (see Fig. 5b). We note here that, while large voltages above 10 V were applied, the actual tuning range restricts to a range of 3~5 V only, and the LC switching seems to saturate for voltage values on the order of 6 ~ 8 V. Larger voltage values were applied only for the purpose of exploring the possible upper-limit of the device driving, for which possible damage of the LC can be expected. Excitation of particular resonant modes under vertical LC switching is analyzed for this case in Supplementary Figure S6. For the 0° and 90° polarization incidence, the tuning range becomes limited with only slight blue shift of the electric resonance from *λ* ≈ 664 nm to *λ* ≈ 662 nm and magnetic resonance red shift from *λ* ≈ 682 nm to *λ* ≈ 688 nm (see Fig. 5a, c). As discussed in Fig. 4b and Supplementary Figure S4, for the *φ* = 45° polarization incidence, the electric resonance at λ ≈ 675 nm for 0 V corresponds to the dominant contribution of electric dipole aligned along the *x*-axis direction (*E*_x_) for 30° LC alignment. Similar to the case of plasmonic particles, the electric dipole resonance in a dielectric particle blue-shifts (red-shifts) when there is a decrease (increase) in the refractive index of the surrounding media (as extensively reported in the literature and widely used for sensing). Thus, the blue shift of this resonance at applied voltage can be attributed to the decreased refractive index of LC environment from *n*_eff_ to *n*_o_ near the nanostructures. The magnetic resonance shift is small for all incident polarization directions and all applied voltages due to its lower sensitivity to the environmental change, as it mainly depends on the wavelength inside the material and the physical size of the nanoparticle^48^. For the 0° and 90° polarization incidence, the electric resonance at *λ* ≈ 664 nm for 0 V corresponds to the dominant contribution of electric dipole aligned along the *y*-axis direction (*E*_y_), with a limited refractive index change resulting in a weak resonance shift. The observed spectral response can be continuously modulated in the range from 3 to 5 V. The high transmission efficiency modulation as much as 65% at the resonance positions (*e*.*g*. the *λ* ≈ 682 nm resonance for 45° incident polarization) can be observed from OFF (~15%) to ON state (~80%). For voltages above 5 V, the resonance positions are almost unchanged in all cases, showing an almost saturation of vertical LC alignment providing ordinary refractive index *n*_o_ for all in-plane incident polarizations.

Note that, despite the difference in the static dielectric permittivity between the LC (for E7 *ε*_par_ ≈ 17, *ε*_per_ ≈ 6) and the TiO_2_ (for amorphous *ε* ≈ 86), that could lead to the distortion in the electric potential distribution around the nanostructures, this effect is in fact minimized for sufficiently small gaps (see Supplementary Figure S8). Nevertheless, LC switching within the array area requires higher voltages compared to the bare LC. This is a consequence of the smaller electrical potential gradient observed in the nanoantenna gaps, as compared to the LC background. The reader can refer to Fig. 5 and Supplementary Figure S2 for a direct comparison of the switching voltage threshold with and without nanostructures and Supplementary Figure S8 for the simulated electric potential distribution in the arrays under 3 V within the tuning range.

## Conclusion

In summary, an electrically tunable all dielectric TiO_2_ metasurfaces designed for visible light modulation (660 ~ 690 nm) is systematically studied. Highly efficient tuning is achieved via electrical switching with a wide tuning range under small driving voltages, in the range of 3 ~ 5 V, together with a large transmission modulation of up to 65%. The analysis of the numerical simulations and experimental results indicates a strong influence of the LC anchoring effect for the thin LC cell considered (1.5 micrometers), induced by the presence of the nanostructures. A preferential alignment with a 30° in-plane deviation with respect to the preset photoalignment direction was found and explained based on the Frank-Oseen model. The anisotropy induced by the LC birefringence needs to be considered to explain the observed mode splitting of electric and magnetic dipole resonances. A wide tuning range of the resonance positions of up to 20 nm was demonstrated for various incident polarizations. This tuning performance can be further improved by better aligning the LC to maximize the refractive index change when light is polarized along the metasurface periodicity. The proposed designs allow dynamic tailoring of resonance positions, which can be used for light modulation, enabling potential application such as dynamic holography. Such LC-based tunable metasurface devices are, moreover, compatible with the current industrial LC technology, and thus realistically close to practical implementation.

## Methods

### Numerical simulation

Numerical simulations were done using a finite-difference time-domain (FDTD) based commercial solver, Lumerical Inc. In the simulations, we consider one single unit-cell containing a disk of TiO_2_ (with a refractive index given by actual sample ellipsometry measurements) to which we applied periodic boundary conditions. The lattice size is *P* = 360 nm in both the x- and y-directions. The top and bottom glasses were modelled as semi-infinite media and the ITO layer (20 nm) was included in the simulations. The E7 LC used in the experiments was modelled as an anisotropic medium with the material parameters taken from the vendor’s specifications (Merck). The LC orientation is defined by the in-plane azimuthal angle *φ* and the polar angle *θ*, the case with *θ* = 90° representing the case in which no voltage is applied and that with *θ* = 0° the case when the LC is fully switched by the external voltage.

### Sample fabrication

The TiO_2_ nanodisk metasurface was fabricated using e-beam lithography from a 190 nm thick TiO_2_ film deposited on a *soda lime* glass coated with ITO (ITO thickness *t* ≈ 20 nm). The TiO_2_ nanodisk arrays have a size of 50 × 50 µm^2^. The nanodisks are arranged in a square periodic lattice with a pitch of *P* = 360 nm and have a diameter of *D* = 320 nm. The TiO_2_ amorphous layer was deposited on the surface of ITO glass by the ion assisted deposition system (Oxford Optofab 3000). Then, a thin layer of Cr (thickness *t* ≈ 30 nm) and polymethyl methacrylate (PMMA) e-beam resist film were sequentially deposited on top of the TiO_2_ film. The structures were then patterned by electron beam lithography (EBL, ELS-7000) to the resist layer. The nanodisks were etched using a reactive ion etching process (ICP-Fluorine, Oxford OIPT Plasmalab System 100) followed by cleaning with oxygen plasma and dipping in an etchant solvent to remove the remaining PMMA and Cr masks.

The photoalignment solution was prepared with a 1% *wt* azobenzene sulfuric dye SD1 dissolved in 2-ethoxyetanol chemical. Then the solution was coated onto another piece of soda lime ITO glass (1.5 × 2 cm^2^) at 5000 rpm for 30 s with the thickness measured being thinner than 50 nm. After the baking process at 60 °C for 30 min, a 45-min exposure under a linearly polarized 450 nm LED blue light was conducted to generate the photo-alignment direction, defined perpendicularly to the incident polarization direction^47^. 1.5 µm silica spacer spheres were uniformly sprayed onto the glass containing the metasurface. The areas containing the nanostructures were sheltered beforehand to prevent the deposition of spacer spheres onto those areas. The two pieces of glass were then bonded and put under 120 °C hot-press for 20 min to fix the sealant glue. To prevent short circuits at the edges of the ITO glasses in the thin gap, the ITO layer on the surface of glass was chemically etched to a smaller square (0.8 × 0.8 cm^2^). Commercial E7 LC (Merck Co., Ltd.) was infiltrated into the 1.5 µm thick LC cell at room temperature in vacuum atmosphere. It was pumped below 5 Pa and post heated at 90 °C for 10 min to help a completed infiltration of LC into the nanostructures. The whole process of LC infiltration was carried out in a cleanroom environment.

### Optical Measurements

The sample was illuminated using a halogen lamp, focused through a dark-field Nikon 5x objective lens with a numerical aperture NA = 0.15, giving a close to normal incidence. A linearly polarized wave with the desired orientation of electric field was obtained using a rotatable wire-grid polarizer (Thorlabs Visible Wire Grid Polarizers, extinction ratio above 800:1 within the 420~700 nm wavelength range). The transmittance was obtained by normalizing the transmission intensity to that of the light source, both detected using a spectrometer (Andor SR-303i) with a 404 × 1600-pixel EMCCD (Andor Newton) attached to the microscope setup.

## Supplementary information

Supplementary Information
