# Supplementary material for: Efficient visible light modulation based on electrically tunable all dielectric metasurfaces embedded in thin-layer nematic liquid crystals
Source: Sci Rep. 2019 Jun 17;9:8673. doi: 10.1038/s41598-019-45091-5 (PMC6572778; doi:10.1038/s41598-019-45091-5)
Supplement: Supplementary file 1 — Supplementary Information [file 41598_2019_45091_MOESM1_ESM.docx]

Supplementary information - Efficient visible light modulation based on electrically tunable all dielectric metasurfaces embedded in thin-layer nematic liquid crystals

Mingyu Sun^1, 2^, Xuewu Xu^2^, Xiao Wei Sun^3^, Xin’an Liang^2^, Vytautas Valuckas^2^, Yuanjin Zheng^1,^ *, Ramón Paniagua-Domínguez^2,^ *, Arseniy I. Kuznetsov^2,^ *

^1^School of Electrical and Electronic Engineering, Nanyang Technological University, 50 Nanyang Avenue, 639798, Singapore

^2^Institute of Materials Research and Engineering, A*STAR (Agency for Science, Technology and Research), 2 Fusionopolis Way, 138634, Singapore.

^3^Department of Electrical and Electronic Engineering, College of Engineering, South University of Science and Technology of China, 1088 Xue-Yuan Road, Shenzhen, Guangdong Province, 518055, China

*Address correspondence to:

[yjzheng@ntu.edu.sg](mailto:yjzheng@ntu.edu.sg)

[ramon_paniagua@imre.a-star.edu.sg](mailto:ramon_paniagua@imre.a-star.edu.sg)

[arseniy_kuznetsov@imre.a-star.edu.sg](mailto:arseniy_kuznetsov@imre.a-star.edu.sg)

Supplementary Figure S1. Calculated electric and magnetic dipole resonance with the varied LC alignment. (a) The electric dipole response for incident polarization varied from *ϕ* = 0^o^ to 90^o^ with LC in-plane alignment set along *x*-axis. (b) Comparison between the electric dipole components along *x* and *y* directions for the same conditions as in (a). (c) The magnetic dipole response for incidence polarizations varied from *ϕ* = 0^o^ to 90^o^ with LC in-plane alignment set along *x*-axis. The intensity of the dipole resonance was normalized by the incident fields.

Supplementary Figure S2**.** Measured transmission of the fabricated LC cell. (a) Transmission of the bare LC cell without metasurface for incidence polarization varied from *ϕ* = 0º to 90º with respect to the photo-alignment direction (*x*-axis). The spectra are detected through a polarizer oriented perpendicular to the incident polarization. This configuration allows measuring a cross-polarized transmission induced by LC birefringence. The transmission signal is maximized for 45^o^ and is almost zero for 0^o^ and 90^o^ polarization incidence indicating a well aligned LC orientation along the photo-alignment direction. (b) Transmittance percentage of the bare LC cell under electrical voltages from 0 to 8V with the photoalignment direction 45^o^ to the polarizer. (c) Captured LC cell image under 0, 3, 4 and 5V with transmission passed through polarizers and analyzer before and after the cell set perpendicular to each other (cross-polarized light detection). The incident polarization is rotated 45^o^ with respect to the LC photo-alignment direction (white arrow). The transmission can be almost blocked above 3V indicating a complete switching of LC molecules to the vertical orientation (along *z*-axis). The measurement configuration is the same as in (b).


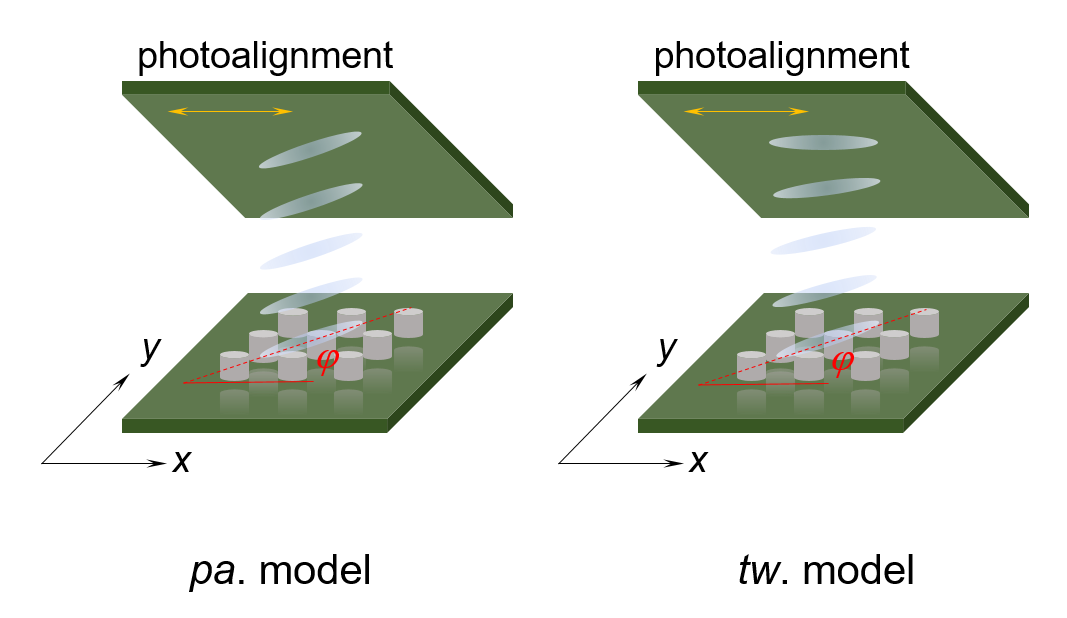


Supplementary Figure S3**.** Sketch of parallel (*pa*.) and twisted (*tw*.) model of LC alignment. *ϕ* angle represents the in-plane LC alignment near the nanoantennas influenced by the anchoring effect.

Supplementary Figure S4. Calculated transmittance percentage for the (a-c) parallel (*pa*.) and (d-f) twisted (*tw*.) model of LC alignment for incident polarization along *ϕ* = 0^o^, *ϕ* = 45^o^ and *ϕ* = 90^o^ directions. The alignment angles are defined by the LC molecules orientation angles near the nanostructures from 0 to 45 degrees representing the influence of the anchoring effect. The data is given in percentage (%).


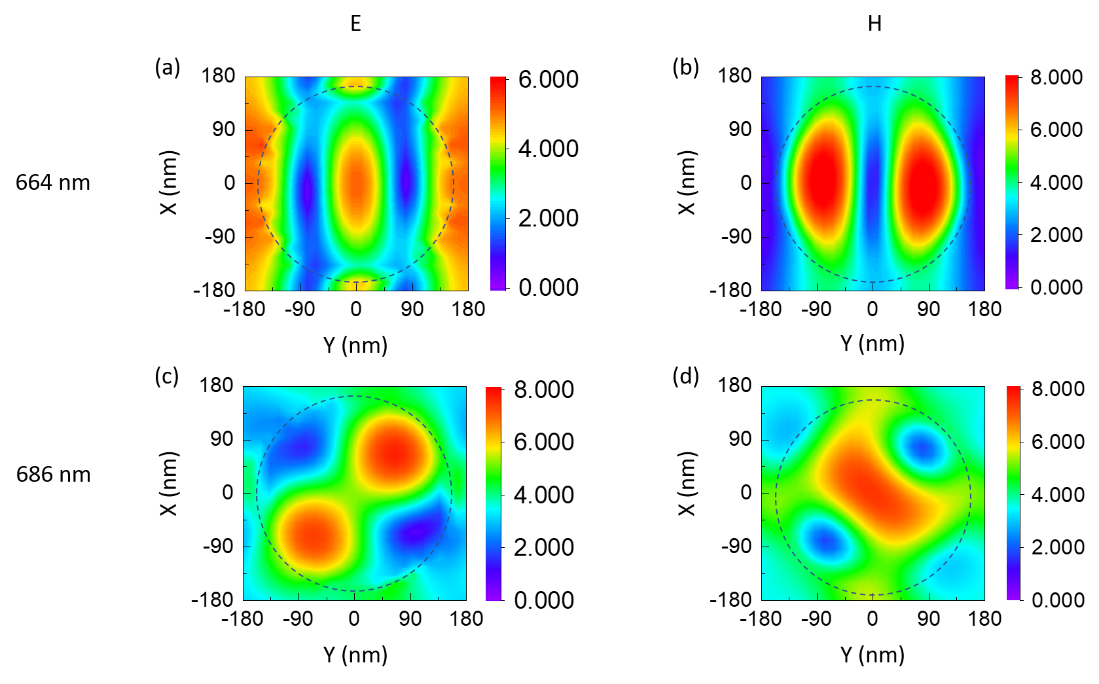


Supplementary Figure S5. Calculated near-field distribution for (a) electric and (b) magnetic mode of λ = 664 nm, and (c) electric and (d) magnetic mode of λ = 686 nm resonances distributed within the *xy* plane. The model is set with *ϕ* = 30^o^ LC in-plane alignment infiltration and *ϕ* = 0^o^ (*x*-axis) incident polarization. The dash circle lines sketched the area of nano disks.

Supplementary Figure S6. Calculated electric and magnetic dipole resonances in the parallel model for the varied incident light polarizations with LC alignment set at 30^o^ with respect to *x*-axis. (a) Electric dipole response for the incidence polarization varied from *ϕ* = 0^o^ to 180^o^ with respect to *x*-axis; (b) Comparison between the excited electric dipole components along *x* and *y* directions; (c) magnetic dipole response for the incidence polarization varied from *ϕ* = 0^o^ to 180^o^ with respect to *x*-axis. The intensity of the dipole resonance was normalized by the incident fields.

Supplementary Figure S7**.** Calculated excitation of (i) the electric, (ii) magnetic dipole resonances, and the electric dipole components along (iii) *x* and (iv) *y* axes direction for LC polar alignment parallel to *θ* = 0^o^ and 90^o^, mimicking the ON (*θ* = 0^o^) and OFF (*θ* = 90^o^) states of the device. The incidence polarization is 0 degree with respect to the x-direction. LC is initially set *ϕ* = 30^o^ in-plane. The intensity of the dipole resonance was normalized by the incident fields.


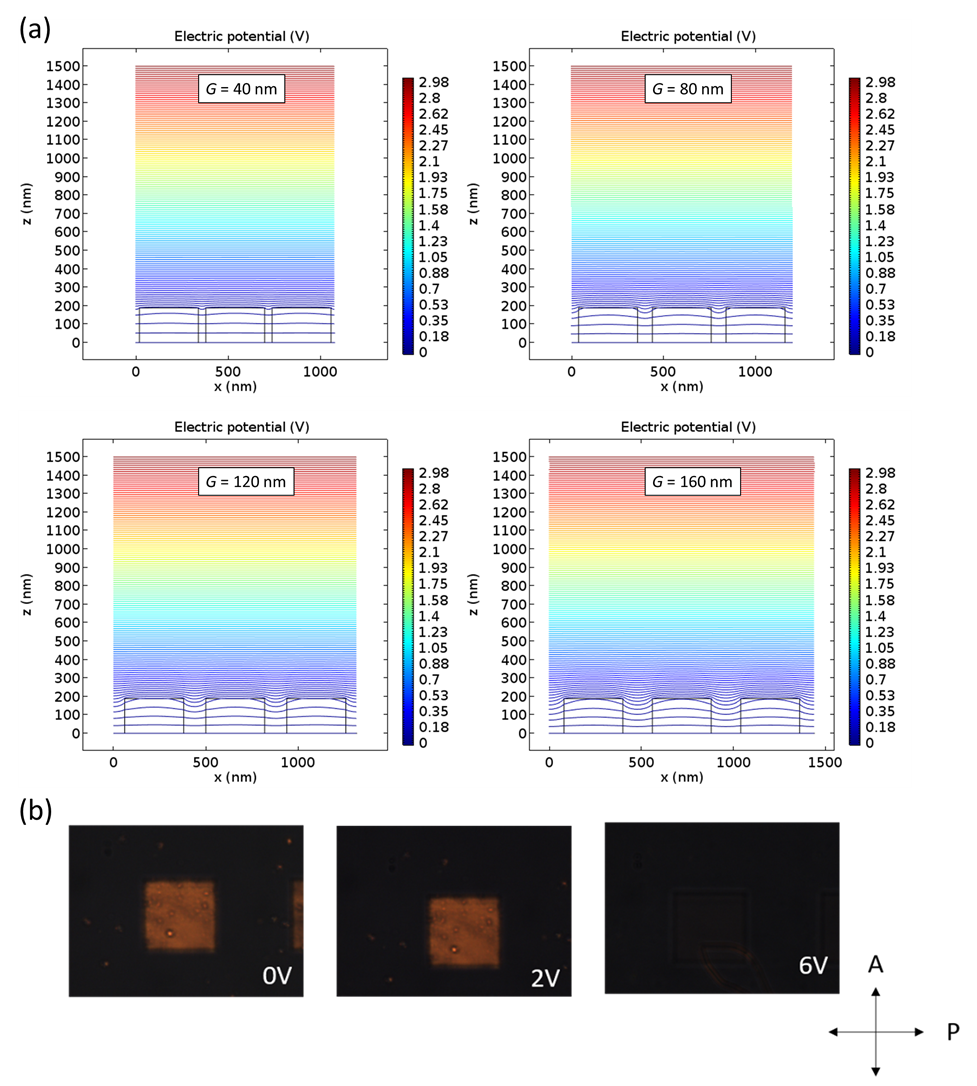


Supplementary Figure S8. (a) Calculated electric potential distribution in LC-embedded TiO_2_ nano-disks under 3V applied voltage for different inter-particle gaps G. The particle radius is 160 nm and the height is 188 nm. The LC thickness is 1.5 um. Only three unit cells are shown here. As seen here, only for gaps above 80 nm a noticeable inhomogeneity in the electric potential distribution is observed in the region between the disks. (b) Microscope images under cross-polarization of a fabricated array captured under 0V, 2V and 6V applied voltage. The arrows define the direction of the polarizer and analyzer. Images show a coherent change of the transmission brightness, pointing at a nearly homogenous, in-plane LC alignment within the area of the array, up to the resolving capabilities of the microscope.
